# Longitudinal Fruit and Vegetable Sales in Small Food Retailers: Response to a Novel Local Food Policy and Variation by Neighborhood Socioeconomic Status

**DOI:** 10.3390/ijerph17155480

**Published:** 2020-07-29

**Authors:** Megan R. Winkler, Kathleen M. Lenk, Darin J. Erickson, Caitlin E. Caspi, Melissa N. Laska

**Affiliations:** 1 Division of Epidemiology and Community Health, University of Minnesota School of Public Health, Minneapolis, MN 55455, USA; lenk@umn.edu (K.M.L.); erick232@umn.edu (D.J.E.); nels5024@umn.edu (M.N.L.); 2 Department of Family Medicine and Community Health, Program in Health Disparities Research, University of Minnesota Medical School, Minneapolis, MN 55455, USA; cecaspi@umn.edu

**Keywords:** healthy food availability, fruits and vegetables, corner stores, store managers, customer purchases, food policy, neighborhood socioeconomic status

## Abstract

Small food retailers, including corner/convenience stores, pharmacies, gas-marts, and dollar stores, have historically stocked limited fruits and vegetables, though this may be changing. We examined increases in sales, customer purchasing, and stocking of fresh and/or frozen fruits and vegetables in small food stores over time and in relation to: (a) a local food policy (the Minneapolis Staple Foods Ordinance) and (b) neighborhood socioeconomic status (SES). We used longitudinal data (2014–2017) from 147 randomly-sampled stores in Minneapolis/St. Paul, USA, collected using interviewer-administered manager surveys (measuring sales and stocking) and customer intercepts/observations (measuring purchasing, *n* = 3039). The local policy required Minneapolis stores to meet minimum stocking standards for fresh/frozen produce and other healthy foods. No ordinance existed in St. Paul. Mixed regression models examined overall change over time and change by city and neighborhood SES. We observed significant increases over time (*p* < 0.05) in sales and purchasing of fresh fruit and in stocking of fresh fruit, frozen fruit, and frozen vegetables. We did not identify consistent statistical evidence for differential change in sales, purchasing, or stocking by city or neighborhood SES. Key study findings suggest limited differential effects of the local ordinance and/or neighborhood SES. However, findings also indicate significant time trends for some products, including consistent improvements in sales, customer purchasing, and stocking of fresh fruit. Given the ready-to-eat convenience of many fresh fruits and their broad appeal, fresh fruit appears a promising target for advancing the healthfulness of small food retailers.

## 1. Introduction

Fruits and vegetables are important aspects of a healthy diet [1]. However, fresh and frozen produce are often not readily available in small food retailers, including corner stores, convenience stores, pharmacies, gas-marts, and dollar stores. These small stores have historically carried a host of unhealthy foods with limited or no healthy options [2,3,4,5,6]. In response, there has been a growing movement to improve the availability of healthy foods in these settings, primarily through voluntary programs or interventions [7] and more recently through policy efforts (e.g., minimum stocking standards) [8,9].

Store managers are important stakeholders in the efforts to improve the healthfulness of food retail settings [10,11]. Much of the success and sustainability of any voluntary or enforced change depends on these important gatekeepers, as they often are responsible for implementing changes in the store. When managers do not believe proposed changes are feasible or worthwhile (e.g., costs outweigh revenue), then efforts aiming to improve healthy food availability may fail [11,12]. Such managerial perspectives are particularly salient for produce in small food stores, as there can be significant challenges to stocking fruits and vegetables and storeowners can be skeptical about their ability to sell such products [13,14,15,16].

Among the many cited barriers to stocking healthier products, such as limited refrigeration and lack of suppliers [13,17], manager perceptions of consumer demand may be one of the most important [11,15,16,18]. Evidence consistently suggests that store managers perceive that consumers favor unhealthy over healthy food and beverages [12,13,15,16,17,18,19,20]. Some managers report this is due to consumers perceiving healthy products to be more expensive [21] or less convenient [15], while others report lack of customer knowledge or interest to improve diet behaviors [12,17,21]. Such unfavorable perceptions about consumer demand for healthy products can inhibit efforts to improve availability in these settings. 

Additionally, store manager perceptions may or may not reflect actual customer demand. For instance, while manager perceptions of product sales may be influenced by customer purchasing, customer purchasing can only occur if the product is stocked in the store. In addition, the likelihood of a manager continuing to stock a product is likely influenced by the managers’ perception of product sales. As such, manager-perceived sales and stocking, as well as observed customer purchasing, are all important to consider when examining patterns of healthy food supply and demand in small food stores.

The purpose of this study was to understand how manager-perceived sales, observed customer purchasing, and manager-reported stocking of fresh and frozen fruit and vegetables changed over four annual assessment periods (2014–2017). We examined change over time in relation to two exposures with key policy and equity implications: (1) a local food ordinance and (2) neighborhood socioeconomic status (SES). The local food ordinance, the Minneapolis Staple Foods Ordinance, was the first of its kind in the U.S., and required all grocery-licensed vendors in Minneapolis, MN, USA, to carry a minimum stock of healthy, staple foods. In 2014, the ordinance was revised to better align with the Dietary Guidelines for Americans 2010 [22] and established minimum requirements for 10 different food/beverage product categories, which have been previously described [9]. Implementation of the revised ordinance occurred in April 2015, and as part of the requirements, vendors were required to stock 30 pounds or 50 items of fresh and/or frozen fruits/vegetables in at least seven varieties, with five or more varieties being fresh. Our prior studies examining the ordinance effect demonstrated that small food stores had varying degrees of increasing compliance and customers showed no clear improvements in purchasing healthier products [9,23]. However, we know little about the ordinance’s effect on manager-perceived sales during this time, especially among specific products, like fresh fruits and vegetables, which are notoriously challenging to stock [13,14,17]. Further, stakeholder discussions prior to the revised ordinance suggested store managers in low SES neighborhoods were especially concerned that their customers would not purchase the required healthful products. Such manager perceptions have been identified in other studies [15,24,25] and are important to examine as they may contribute to an unintended consequence of worsening disparities in low SES communities if such perceptions prevent improvements in fruit and vegetable availability. 

## 2. Materials and Methods 

### 2.1. Study Design 

Data for this analysis were part of the larger STORE (STaple foods ORdinance Evaluation) study. The primary objective of STORE was to assess the effects of the Minneapolis Staple Foods Ordinance, including overall changes in compliance, the healthfulness of the store environments, and customer purchasing in small food stores [9]. Data were collected in Minneapolis as well as in an adjacent city, St. Paul, Minnesota, USA, which served as the study’s comparison (i.e., control) site, across four time points—pre-ordinance revisions in Fall 2014 and three post-implementation time points, including Fall 2015 (ordinance revisions activated, no enforcement), Summer 2016 (early enforcement begins), and Fall 2017 (continued monitoring and enforcement). As previously described [9], stores were randomly selected based on administrative lists of licensed retailers. Stores were excluded from selection if they were supermarkets, authorized as a US Special Supplemental Nutrition Program for Women, Infants, and Children (WIC) store, and/or a retailer exempt from the ordinance in Minneapolis, as well as comparable stores in St. Paul. Of the 180 stores (90 Minneapolis, 90 St. Paul) sampled, 23 stores were deemed ineligible after a pre-data collection store visit and two stores did not provide consent, resulting in 155 stores that consented and participated at one or more study time points (See Supplemental Figure S1). The University of Minnesota Institutional Review Board approved all study protocols involving human subjects prior to data collection (1311S45924). 

#### 2.1.1. Data Collection

At each time point, teams of two data collectors visited stores primarily on weekdays between 10am and 7pm. Store owners or managers were asked to participate in an interviewer-administered manager survey, and customer intercepts were performed with manager permission to evaluate customer purchasing. We conducted intercept interviews with customers exiting stores and recorded observed food and beverage purchases (details on data collection methods, participant eligibility, and participant response rate have been published elsewhere) [9,26]. Interviewer-administered manager surveys were conducted in the store at a time convenient for the manager [27]. 

#### 2.1.2. Sample

The sample for this analysis included stores that had a manager participate at one or more time points (*n* = 147 stores) and/or stores that had customers that were interviewed at one or more time points (*n* = 147 stores; *n* = 3039 customer interviews conducted across 2014–2017; See Supplemental Figure S1). Collectively, 137 stores had both manager and customer-level data. As previously reported [27], participating managers self-reported being predominantly male, non-Hispanic White, in their late thirties, and on average had managed and/or owned the store for approximately four years. Customers participating in intercept interviews self-reported being predominantly male, non-Hispanic White or non-Hispanic Black, approximately 40 years old, employed, and shopped at the store at least weekly [9].

### 2.2. Measures

#### 2.2.1. Manager-Perceived Increases in Sales and Reported Stocking

We examined eight outcomes reported by managers regarding perceived increases in sales and stocking for fresh and frozen produce. Manager-perceived increases in sales were measured from a series of items asking the store manager, “Over the past 6 months, did sales of [product] decrease, stay the same, or go up,” which was asked separately for fresh fruit, fresh vegetables, frozen fruit, and frozen vegetables. Response options included “decreased,” “stayed the same,” “increased,” or “not offered,” and we dichotomized responses to “increased” versus all other responses. We operationalized manager-reported stocking with the same item series, and dichotomized responses using the “not offered” versus all other responses. 

#### 2.2.2. Observed Customer Purchasing

Data on foods and beverages purchased by customers were entered by trained staff into the Nutrition Data System for Research (NDSR), a software application developed at the University of Minnesota Nutrition Coordinating Center that generates values for nutrients and food servings for numerous product categories [28]. We identified customers at each time point who purchased at least ½-serving (i.e., ¼ cup) of fruit or vegetables. We selected a ½-serving to ensure products that were <1 serving (e.g., lime, garlic head, kiwi), but likely considered by managers as a fresh produce stocked or sold in the store, were included. Then, two study team members reviewed all customer purchases and coded them as: fresh fruit, fresh vegetable, frozen fruit, and frozen vegetable (See Supplemental File S1). Like the manager-perceived outcomes, we operationalized customer purchasing at the store level by dichotomizing stores into those who had at least one customer purchase of a ½-serving of the product versus stores that did not. Purchasing of frozen fruit and frozen vegetables was only observed at one time point, and thus, we could only examine customer purchasing over time of fresh fruit and fresh vegetables. 

#### 2.2.3. Local Food Ordinance

As the ordinance was in effect only in Minneapolis, we examined its impact by comparing outcomes across the two cities—Minneapolis and St. Paul, MN, USA. Minneapolis and St. Paul have similar population demographics [9] and are located immediately adjacent to each other. 

#### 2.2.4. Neighborhood Socioeconomic Status (SES)

Neighborhood data were obtained from five-year American Community Survey estimates (2009–2015) [29] and were attributed to stores based on census tract location. Store census tracts were classified into low SES or higher SES, regardless of the city in which they were located. Following our prior work [23], we defined low SES as census tracts with >50% of residents at or below 185% of the U.S. federal poverty level, based on household income and size.

### 2.3. Analysis

We performed descriptive analyses on stores with managers who participated in at least one study time point (*n* = 147).

#### 2.3.1. Testing Effects of Local Food Ordinance

We computed mixed model logistic regression analyses to test the effect of the local food ordinance on all manager-perceived sales, observed customer purchasing, and manager-reported stocking outcomes. We first tested an overall time by city interaction (df = 3) to examine the effect of the revised ordinance over all four time points. We then explored secondary analyses of single degree of freedom planned contrasts to test time by city interactions from baseline to different phases of ordinance implementation (i.e., from time 1 to 2, from time 1 to 3, and from time 1 to 4). Several managers (e.g., job title, gender) and customer characteristics (e.g., age, employment status) were examined for their potential to confound the associations, and none were identified to have an association with both cities and any of the applicable outcomes (data not shown). Thus, final models were adjusted for repeated measures only, and we report the estimated prevalence and standard errors of stores for each outcome over time by city.

#### 2.3.2. Testing Change over Time by Neighborhood SES 

Results from the ordinance models informed our analytic approach for neighborhood SES. From this and other studies [9,27], we have found consistent evidence of secular changes (change over time) in various outcomes though minimal evidence of the ordinance contributing to those changes (i.e., few significant differential changes over time by city)—attributed to the persistent low compliance in Minneapolis by 2017 [9]. As such, we chose to focus on secular changes by SES over time in data from both cities aggregated together. After inspection of the data, we decided that a linear model with time treated as a continuous measure would capture the most variability. Using mixed model logistic regression analyses, we examined the main effects for time (continuous, df = 1) and neighborhood SES (df = 1), as well as an overall time by SES interaction (df = 1) on all manager-perceived sales increases, observed customer purchasing, and manager-reported stocking outcomes. All models were adjusted for repeated measures as well as city to control for the study sampling design. To assist with interpretation, we also report the predicted prevalence of stores for each outcome over time by low and higher neighborhood SES.

## 3. Results

### 3.1. Descriptive Characteristics of Small and Non-Traditional Food Stores

Characteristics of the stores are presented in Table 1. Most were either a gas-mart (38%) or corner/convenience store (37%). Half were corporate- or franchise-owned (54%), nearly all (94%) were authorized through the U.S. Supplemental Nutrition Assistance Program (SNAP), and less than one-third were located in low SES neighborhoods (29%).

### 3.2. Impact of Local Food Ordinance

Table 2 presents changes in manager-perceived increases in sales, observed customer purchasing, and manager-reported stocking across time in Minneapolis and St. Paul. There was a significant increase from 2014–2017 in reported stocking of fresh fruits, frozen fruits, and frozen vegetables, with estimated prevalence increasing in both cities by at least 10 percentage-points. There was also a significantly greater proportion of managers in Minneapolis compared to St. Paul reporting they stocked fresh fruit, fresh vegetables, and frozen vegetables; however, there was no significant differential change over time by city, suggesting no impact of the ordinance on reported stocking of these products.

For manager-perceived increases in sales, fresh fruit was the only outcome with a statistically significant change over time (*p* < 0.001). Like reported stocking, there was a higher predicted prevalence of managers in Minneapolis compared to St. Paul perceiving increases in sales for fresh fruit, fresh vegetables, and frozen vegetables, but no evidence in change over time by city.

For observed fresh fruit purchasing, we found a statistically significant interaction between time and city, suggesting the prevalence of stores with at least one customer purchase of fresh fruit increased more in Minneapolis compared to St. Paul. Change by city was most pronounced at the phases of activation of the ordinance revisions (2015, *p* = 0.01) and continued monitoring (2017, *p* = 0.04), though directionality of city changes were not consistent across all time points. We observed no statistically significant differences in the proportion of stores with a customer purchase of fresh vegetables over time, by city, or over time by city.

### 3.3. Neighborhood SES

Table 3 presents changes in manager-perceived increases in sales, observed customer purchasing, and manager-reported stocking across time by neighborhood SES. We again observed increases in reported stocking over time, which were statistically significant for fresh fruit, frozen fruit, and frozen vegetables; however, there were no significant differences by neighborhood SES or over time by neighborhood SES.

We also observed statistically significant increases over time in the proportion of managers perceiving better sales for fresh fruit and frozen fruit. Estimates show that while more managers in low versus higher SES communities perceived recent increases in sales for all products at baseline, over time, the proportion of managers in higher SES neighborhoods perceiving increases rose while the proportion in low SES neighborhoods remained the same or fell. However, such changes by SES were only statistically significant for frozen vegetables (*p* = 0.02).

We also observed that the proportion of stores with a customer purchase of fresh fruit significantly increased over time (*p* = 0.04) by approximately 10 percentage-points in both higher and low SES neighborhoods, and, like most other outcomes, we did not observe statistically significant differences by SES or over time by SES.

## 4. Discussion

In this study, we examined how manager-perceived sales, observed customer purchasing, and manager-reported stocking of fresh and frozen fruits and vegetables changed over time and in response to two exposures—a local food ordinance and by neighborhood SES. Key findings include: (a) limited evidence of a beneficial effect from the local food ordinance on sales, purchasing, and/or stocking; and (b) no evidence of worsening SES disparities in stocking and purchasing over time. A third key finding was the significant time trends we observed for sales, purchasing, and/or stocking of several products; however, fresh fruit was the only product that displayed consistent improvements in all three of these outcomes.

Examining change in response to the Minneapolis Staple Foods Ordinance demonstrated that there was not a clear impact of the revised ordinance on manager-perceived sales, manager-reported stocking, or customer purchasing of fresh or frozen fruits and vegetables. The lack of change in manager-perceived sales differs from prior research, which suggests manager perceptions of sales often increase following store-based interventions or revisions to national nutrition programs for healthy foods [18,19,30,31]. However, we also did not identify a significant effect of the ordinance on manager-reported stocking of products—a finding that matches our objective assessments of store compliance with the fruit and vegetable requirement [9]. Customer purchasing of fresh fruit was the only outcome that displayed a statistically significant change over time by city, though this should be interpreted with caution, given (a) the directionality of city changes was inconsistent across time, and (b) this may primarily be attributed to the very low rates in Minneapolis observed in 2014 (pre-ordinance). Together, these findings could suggest that a simple policy, such as the Minneapolis ordinance, may not be sufficient to improve the availability of fruits and vegetables in small food retailers, particularly over this relatively short study period. Policy approaches recommended by the World Health Organization and others [32,33], coupled with the findings from this and other work [9,23], suggest more comprehensive strategies may be necessary to improve the healthfulness of retail settings. Promising additional strategies include things like incentivizing retailers and facilitating product distribution. In addition, evaluating outcomes over a longer time horizon and understanding the dose-response relationship [34] of store compliance with the ordinance are important ways to extend this work and disentangle a failure of the policy from other explanations, such as implementation and compliance.

Importantly, we did not find evidence of worsening disparities across store neighborhood SES in manager-reported stocking or observed customer purchasing of fresh and frozen fruit and vegetable products from 2014–2017. However, results from manager-perceived increases in sales were more complicated. Patterns suggested store managers in higher SES communities may have perceived better increases in sales over time, while managers in low SES communities had static or worsening perceptions of sales; yet, these only were statistically significant for frozen vegetables. Given these mixed findings, it is difficult to say whether a persistent bias of poor customer fruit and vegetable purchasing does or does not remain among managers of low SES communities, as cited in other investigations [15,24,25] and found in our formative discussions with stakeholders. Fortunately, if a bias does remain, findings suggest that this did not have an impact on stocking or customer purchasing of fruit and vegetable products.

One of the most compelling findings from this study is that sales, stocking and purchasing of fresh fruit all significantly improved over time. Despite having a shorter shelf life, fresh fruit is often a ready-to-eat product, which maps onto to the “convenience-focused” business model of most U.S. small food retailers. Even so, there remains much room for improvement, as estimates indicate that, at best, a fresh fruit purchase was only occurring among 15–25% of stores. Evaluating and adjusting merchandising efforts for fresh fruit, such as placement and appeal, may be one important way retailers could further promote the sales of these items.

In contrast, fresh or frozen vegetables and frozen fruit did not display consistent improvements over time in stocking, sales, or purchasing. These products may not be as well-matched with the convenience business model, as they more often require some at-home preparation. As such, it may be important for small food retailers to reconsider how they can make these products fit into their convenience model, by prepping and packaging products so they can be eaten by hand (e.g., pre-cut carrots, cucumbers, red peppers with dip) and on-the-go (e.g., pre-package personal salad).

### Strengths and Limitations

There were both strengths and limitations to the current study. Two strengths included the longitudinal study design and random sampling of stores, which allowed us to observe trends over time in the supply and demand of fruit and vegetable items among small food retailers. Despite this, data were drawn from one US geographic area and customers and managers were predominantly non-Hispanic and male, which may not be generalizable to other regions. In addition, while we observed differences by city in stocking and manager perceptions of sales at baseline (pre-policy revisions), trends over time did not vary by city. Such findings highlight the importance of employing pre-/post-policy study designs rather than post-only designs, which cannot separate effects of a policy or intervention from other area-specific differences.

An additional strength is that we differentiated between fresh and frozen fruit and vegetable outcomes, which were included in the Minneapolis ordinance and helped capture different degrees of product convenience for consumers. We also used a measure of store neighborhood SES that reasonably approximated low SES communities; yet, additional work is needed to understand if greater variation in neighborhood SES or whether SES of a store’s actual customer base, which is less geographically bound, are more sensitive measures for studying potential disparities in fruit and vegetable availability and sales.

Despite this study being one of the only to have detailed longitudinal data on a sample of small and non-traditional stores, our sample size of 147 stores may have limited our ability detect small effects. We also measured stocking using managers’ self-report, and, despite results mapping onto observed assessments of ordinance compliance [9], managers in Minneapolis may have been more motivated to report stocking to indicate compliance. In addition, while customer purchasing was measured via observed assessments, we operationalized purchasing at the store level using a binary variable. Purchasing data were also collected at stores at different times of the day for only up to a single hour per store, and likely contributed to some of the variation in estimates we observed (e.g., the very low prevalence of fresh fruit in Minneapolis at baseline). Still, customer intercepts remain one of the most feasible approaches to objectively measure purchasing in these small retailers, given many stores do not collect or are hesitant to share detailed sales data.

## 5. Conclusions

Today, consumption of fruit and vegetables remains significantly below recommendations across many countries [35,36,37], and disparities in consumption and availability remain important issues. As such, well-rounded, multipronged solutions are necessary to not only ensure items are available in stores, such as the Minneapolis Staple Food Ordinance, but also to encourage customers to purchase these items. Complimentary policy changes to stocking requirements, such as the fruit and vegetable incentive programming integrated into the U.S. SNAP program (i.e., the Food Insecurity Nutrition Incentive Program [38] or other parallel initiatives [39]) may be one approach, as it could encourage customer purchasing of fruits and vegetables as well as directly support consumers and stores in low SES neighborhoods. Even so, additional system approaches to address the challenges unique to small food stores, such as limited options for produce distribution and store infrastructure, will be essential to ensure the demand, stocking, and manager-perceived sales of fruits and vegetables are also improved in these important food spaces.

## Figures and Tables

**Table 1:** Descriptive characteristics of small and non-traditional stores participating in interviewer-administered manager survey in Minneapolis–St. Paul, MN, USA (*n* = 147).

Characteristics	*N* (%)
Store type	
Corner stores, convenience stores, small grocers	55 (37)
Food-gas marts	56 (38)
Dollar stores	14 (10)
Pharmacies	21 (14)
General Retailers	1 (1)
Corporate status	
Corporate/Franchise-owned	80 (54)
Independently-owned	67 (46)
Store size (no. of cash registers)	
1 register	47 (33)
2–3 registers	68 (47)
4+ registers	29 (20)
SNAP Authorized ^1^	
Yes	138 (94)
No	9 (6)
Neighborhood SES	
Low	42 (29)
High	105 (71)
City	
Minneapolis	84 (57)
St. Paul	63 (43)

^1^ SNAP authorized, store is authorized to accept benefits from customers participating in the US Supplemental Nutrition Assistance Program.

**Table 2:** Impact of a local food ordinance (Minneapolis Staple Food Ordinance) on stocking, manager-perceived increases in sales, and observed customer purchasing of fruit and vegetable products over time (2014–2017) in Minneapolis and St. Paul, MN, USA (*N* = 147 stores).

Data Type	Outcome	City	Assessment Period				Overall Effects		
			2014 Pre-Ordinance	2015 Ordinance Activated	2016 Early Enforcement	2017 Continued Monitoring	Main Effects		Interaction
							Time	City	Time × City
			Predicted % (SE)				P (df = 3)	P (df = 1)	P (df = 3)
	Stocking ^†^								
	Fresh Fruit	Minneapolis	81.4 (5.9)	78.5 (5.1)	90.9 (3.5)	95.3 (2.6)	0.002	0.001	0.12
		St. Paul	62.9 (8.2)	65.1 (7.3)	68.8 (6.7)	73.9 (6.5)			
		*p*-net	--	*p* = 0.57	*p* = 0.36	*p* = 0.17			
M	Fresh Vegetables	Minneapolis	60.5 (7.5)	60.0 (6.1)	63.6 (5.9)	66.2 (5.9)	0.16	<0.001	0.51
A		St. Paul	37.1 (8.2)	32.6 (7.1)	29.8 (6.7)	50.0 (7.4)			
N		*p*-net	--	*p* = 0.70	*p* = 0.35	*p* = 0.59			
A									
G	Frozen Fruit	Minneapolis	41.9 (7.5)	49.2 (6.2)	42.4 (6.1)	65.6 (5.9)	<0.001	0.09	0.75
E		St. Paul	31.4 (7.9)	30.2 (7.0)	31.9 (6.8)	60.9 (7.2)			
R		*p*-net	--	*p* = 0.52	*p* = 1.0	*p* = 0.65			
	Frozen Vegetables	Minneapolis	48.8 (7.6)	52.3 (6.2)	58.5 (6.1)	79.7 (5.0)	<0.001	0.002	0.77
		St. Paul	31.4 (7.8)	25.6 (6.7)	38.3 (7.1)	58.7 (7.3)			
		*p*-net	--	*p* = 0.39	*p* = 0.87	*p* = 0.61			
	Perceived Increases in Sales ^‡^								
R	Fresh Fruit	Minneapolis	32.6 (7.1)	32.3 (5.8)	56.1 (6.1)	34.4 (5.9)	<0.001	0.003	0.23
E		St. Paul	8.6 (4.7)	16.3 (5.6)	29.2 (6.6)	30.4 (6.8)			
P		*p*-net	--	*p* = 0.27	*p* = 0.46	*p* = 0.06			
O									
R	Fresh Vegetables	Minneapolis	25.6 (6.7)	35.4 (5.9)	24.2 (5.3)	29.2 (5.6)	0.09	0.003	0.35
T		St. Paul	5.7 (3.9)	9.3 (4.4)	6.4 (3.6)	17.4 (5.6)			
E		*p*-net	--	*p* = 0.92	*p* = 0.82	*p* = 0.15			
D									
	Frozen Fruit ^1^	Minneapolis	11.6 (4.9)	12.3 (4.1)	12.1 (4.0)	12.5 (4.1)	0.22	0.09	0.31
		St. Paul	0 (0)	4.7 (3.2)	8.5 (4.1)	15.2 (5.3)			
		*p*-net	--	*p* = 0.57	*p* = 0.28	*p* = 0.08			
	Frozen Vegetables ^1^	Minneapolis	16.3 (5.6)	13.9 (4.3)	7.7 (3.3)	12.5 (4.1)	0.88	0.009	0.20
		St. Paul	0 (0)	4.7 (3.2)	6.4 (3.6)	6.5 (3.6)			
		*p*-net	--	*p* = 0.30	*p* = 0.04	*p* = 0.17			
	Customer Purchasing ^§^								
O	Fresh Fruit	Minneapolis	3.1 (2.2)	17.2 (4.7)	11.3 (4.0)	18.3 (5.0)	0.05	0.47	0.03
B		St. Paul	12.8 (4.9)	7.1 (4.0)	23.9 (6.3)	14.9 (5.2)			
S		*p*-net	--	*p*= 0.01	*p* = 0.50	*p* = 0.04			
E									
R	Fresh Vegetables	Minneapolis	7.8 (3.4)	7.9 (3.4)	4.8 (2.7)	13.3 (4.4)	0.37	0.35	0.16
V		St. Paul	12.8 (4.9)	7.1 (4.0)	17.4 (5.6)	12.8 (4.9)			
E		*p*-net	--	*p* = 0.40	*p* = 0.29	*p* = 0.39			
D									

Note: Logistic regression models adjusted for repeated measures over time. *p*-net values refer to changes in time × city effect from 2014 to 2015, 2014 to 2016, and 2014 to 2017, respectively. ^1^ Linear regression model used. ^†^ Percent of managers reporting their store stocks/offers the product. **^‡^** Percent of managers reporting sales of product increased in previous 6 months. ^§^ Percent of stores with at least one observed customer purchase of ½ a serving of product.

**Table 3:** Changes in proportion of stocking, manager-perceived increases in sales, and observed customer purchasing of fruit and vegetable products over time (2014–2017) across neighborhood socioeconomic status (SES) in Minneapolis–St. Paul, USA (*N* = 147 stores).

Data Type	Outcome	SES	Assessment Period				Overall Effects		
			2014 Fall	2015 Fall	2016 Summer	2017 Fall	Main Effects		Interaction
							Time	SES	Time × SES
			Predicted %				P (df = 1)	P (df = 1)	P (df = 1)
	Stocking ^†^								
	Fresh Fruit	Low	69.3	74.8	76.8	80.9	0.002	0.91	0.40
		Higher	70.4	78.0	81.8	87.6			
M									
A	Fresh Vegetables	Low	52.0	59.5	63.1	69.8	0.29	0.93	0.46
N		Higher	44.1	47.7	49.0	52.2			
A									
G	Frozen Fruit	Low	41.7	48.5	52.9	60.5	<0.001	0.24	0.39
E		Higher	28.6	38.0	45.1	57.8			
R									
	Frozen Vegetables	Low	41.3	53.9	61.5	73.8	<0.001	0.63	0.98
		Higher	31.8	43.2	51.1	64.9			
	Perceived Increases in Sales ^‡^								
R	Fresh Fruit	Low	29.3	29.7	28.7	27.9	0.01	0.47	0.16
E		Higher	23.7	30.0	34.2	42.6			
P									
O	Fresh Vegetables	Low	23.4	24.7	24.2	24.2	0.29	0.49	0.47
R		Higher	16.1	18.8	20.0	22.9			
T									
E	Frozen Fruit	Low	16.4	15.9	15.1	14.1	0.03	0.03	0.08
D		Higher	3.6	5.9	8.2	14.3			
									
	Frozen Vegetables	Low	17.1	11.7	8.1	4.5	0.24	0.07	0.02
		Higher	6.0	7.8	8.9	11.5			
O									
B	Customer Purchasing ^§^								
S	Fresh Fruit	Low	13.5	16.6	19.5	25.5	0.04	0.25	0.85
E		Higher	7.4	9.7	11.9	16.4			
R									
V	Fresh Vegetables	Low	6.2	7.7	9.3	12.8	0.52	0.55	0.56
E		Higher	9.6	10.3	10.9	12.0			
D									

Logistic regression models adjusted for repeated measures over time and city. ^†^ Percent of managers reporting their store stocks/offers the product. ^‡^ Percent of managers reporting sales of product increased in previous 6 months. ^§^ Percent of stores with at least one observed customer purchase of at least ½ a serving of product.

## Data Availability

The datasets used for the analyses presented in this manuscript are available online via the Data Repository for the University of Minnesota. The only data withheld from these datasets (i.e., will not be publicly accessible) are variables detailing (1) store type (i.e., convenience store, gas mart, pharmacy, dollar store) and (2) number of cash registers in the store. Concerns have been raised about the ability to deduce which retailers participated if these variables were to be included in the dataset, particularly when available in combination with the neighborhood-level data. These two variables were only used in our descriptive analyses (presented in Table 1), and their omission will not hinder a reader’s ability to replicate the findings from our main models.

## Supplementary Materials

The following are available online at https://www.mdpi.com/1660-4601/17/15/5480/s1, Figure S1: Flow of participants for store manager surveys and customer intercepts at each data collection time point of the study (2014–2017) and File S1: Classifying Customer Fruit and Vegetable Purchases.
