# Supplementary material for: Longitudinal Fruit and Vegetable Sales in Small Food Retailers: Response to a Novel Local Food Policy and Variation by Neighborhood Socioeconomic Status
Source: Int J Environ Res Public Health. 2020 Jul 29;17(15):5480. doi: 10.3390/ijerph17155480 (PMC7432731; doi:10.3390/ijerph17155480)
Supplement: Supplementary file 1 [file ijerph-17-05480-s001.pdf]

Figure S1. Flow of participants for store manager surveys and customer intercepts at each data collection time point of the study (2014-2017)[a]

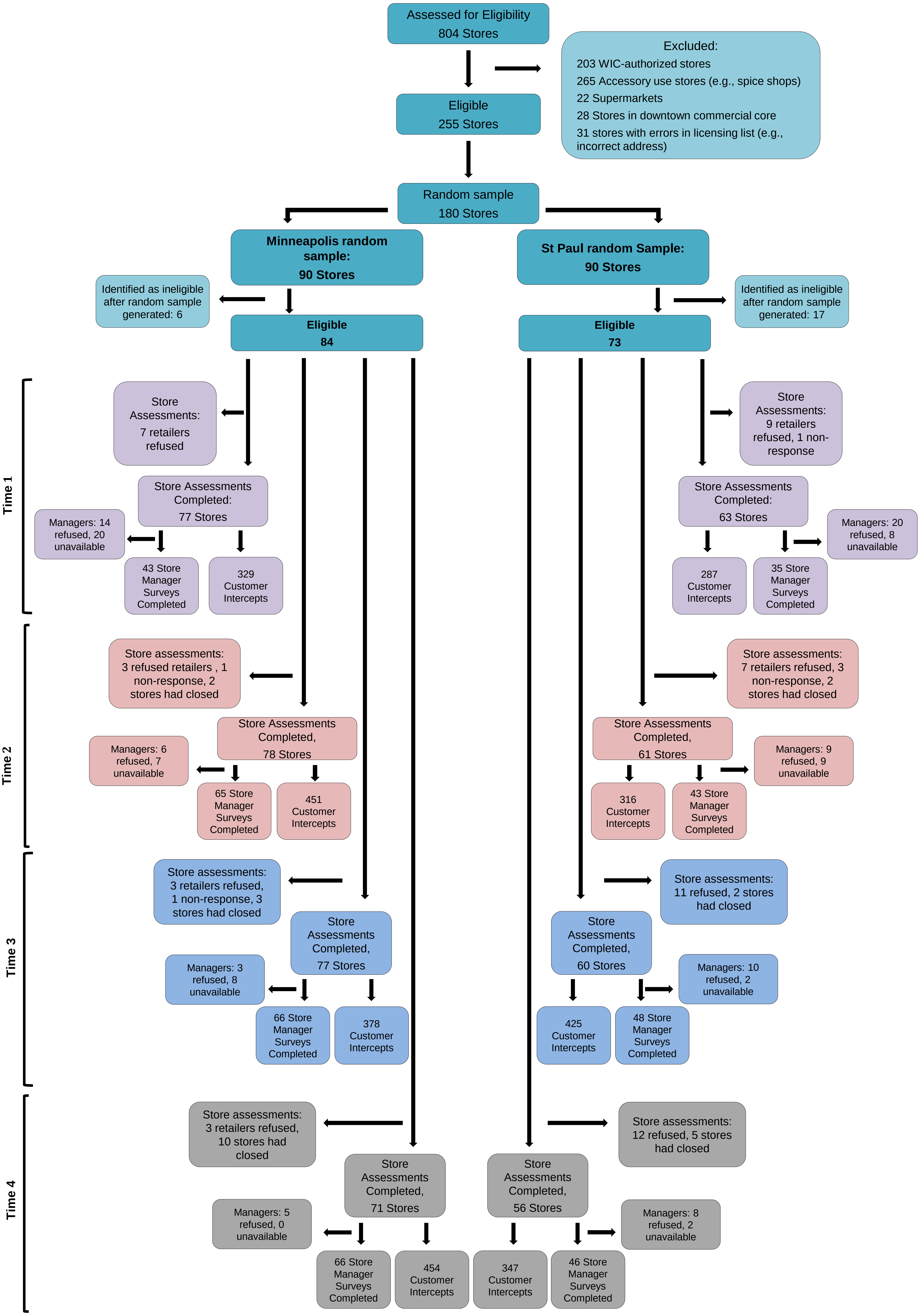

[a] Figure was adapted from a figure previously published by BioMed Central [Laska MN, Caspi CE, Lenk K, Moe SG, Pelletier JE, Harnack LJ, et al. Evaluation of the first U.S. staple foods ordinance: impact on nutritional quality of food store offerings, customer purchases and home food environments. *International journal of behavioral nutrition and physical activity*. 2019;16(1):83.]. Manager surveys and customer intercepts were data sources used in this study. Results from the store assessments are available in [9].

File S1: Classifying Customer Fruit and Vegetable Purchases

Rules for retrieving customer purchases:

1. We used data from Food File 7 of the Nutrition Data System for Research (NDSR):
   a. For fruit purchases:
      i. We identified customers who purchased >=0.5 serving (*at least* ¼ cup) of any of the 3 fruit variables at each time point:
         1. Citrus Fruit
         2. Fruit excluding citrus fruit
         3. Avocado and Similar
      ii. We excluded the 4 variables for fruit juices, fried, and savory snacks as these preparations are assumed to not include a fresh or frozen fruit

   b. For vegetable purchases:
      i. We identified customers who purchased >=0.5 serving (*at least* ¼ cup) of any of the 7 vegetable variables at each time point:
         1. Dark-green vegetables
         2. Deep-yellow vegetables
         3. Tomato
         4. White Potatoes
         5. Other Starchy Vegetables
         6. Other Vegetables
         7. Legumes
      ii. We excluded the 4 variables for vegetable juice, fried, and savory snacks as these preparations are assumed to not include a fresh or frozen fruit

2. We opted for >=0.5 servings (1/4 cup) to ensure we captured products that managers likely counted as a fresh produce purchase but were small in size (e.g., lemons, limes, garlic head, kiwi). While allowing us to capture most fresh products, there are still some that may have not met this threshold (e.g., fresh parsley)

Rules for coding the purchased items:

1. First, we excluded fruit or vegetable items that were part of the following product codes as these would not follow the ordinance requirements of fresh or frozen fruit & vegetables:

   a. Frozen pizzas
   b. Mixed dishes
   c. Fruit smoothies
   d. Canned (e.g., soup, salsa, hummus, baby food, tomato sauce) or dried (e.g., dates, dried cranberries) preparations

2. For remaining products purchased:
    a. We counted only those that were uncooked (unprepared) ***and*** at least 0.5 servings (1/4 cup) of fresh or frozen
        i. Thus we included items such as ready-to-go personal salads, sandwich toppings that were > ¼ cup, guacamole (fresh avocado), caramel covered apples, tuna salad, coleslaw, and tabbouleh salad, as some auditors would count these products as meeting the ordinance standards
    b. We also were required to make some assumptions about preparation based on investigator expertise about the marketplace in these stores (e.g., cooked vegetable preparations are not served); limitations of the NDSR dataset (e.g., a cooked preparation type is the only option for plantains, cassava, and frozen vegetables); and/or how data collectors coded each product (e.g., did not specify a preparation type). These rules included:
        i. All greens (e.g., iceberg lettuce), avocados, vegetable combos (e.g., broccoli, carrots, and cauliflower) and fruits (e.g., pineapple) that did not have a clear or specified preparation were assumed to be fresh, except dates which were assumed to be dried
        ii. All plantains and cassava purchased, which can only be coded in NDSR as cooked, were assumed to be fresh and not cooked
        iii. All vegetable products identified as 'cooked from frozen' were assumed to be frozen and not cooked
        iv. All vegetable products identified as 'cooked from fresh' were assumed to be fresh and not cooked
        v. For any remaining purchases that could not be coded following these rules (n=3 purchased items), hard-copy data collection forms were reviewed and authors discussed and agreed upon categorization
    c. Finally, we counted avocados and guacamole as fresh vegetable purchases
